# One-month early time-restricted eating mitigates brain aging and enhances memory in males with metabolic syndrome: an MRI structural study

**DOI:** 10.3389/fragi.2026.1752738

**Published:** 2026-02-18

**Authors:** Yue Qin, Xiaoshi Li, Lei Wang, FengLin Shuang, Xin Jin, Tingting Qu, Yuying Wang, Yunbing Wu, Juan Tian, Yifan Qian, Qianqian Lv, Zirui Wang, Yarong Wang, Ying Xing

**Affiliations:** 1 Department of Radiology, Xi’an Daxing Hospital Affiliated to Yan’an University, Xi’an, China; 2 Department of Radiology, The First Affiliated Hospital of Xi’an Jiaotong University, Xi’an, China; 3 Department of Endocrinology, Xi’an Daxing Hospital Affiliated to Yan’an University, Xi’an, China; 4 Xi’an Jiaotong University Health Science Center, Xi’an, China

**Keywords:** brain age, cognitive function, early time-restricted eating, metabolic syndrome, voxel-based morphometry

## Abstract

**Background:**

Metabolic syndrome (MetS) has emerged as a global health concern challenges to human brain aging and memory function. While early time-restricted eating (eTRE) has been widely recognized as an effective dietary approach for improving metabolic regulation, its potential influence on brain aging and memory performance in individuals with MetS has not yet been fully elucidated.

**Methods:**

Twenty three males with MetS were enrolled and underwent a 1-month eTRE intervention. Assessments included metabolic profiling, memory evaluation, and MRI scanning at both baseline and post-intervention. Brain age gap (BAG) was estimated using the brainageR package, after which voxel-based morphometry (VBM) was applied to identify gray matter regions whose structural changes contributed most to brain age alterations. Subsequently, correlation analyses were performed to examine the associations between these regional changes and memory function.

**Results:**

After 1-month of eTRE, males with MetS showed a significant reduction in BAG difference (*p* < 0.05), along with improvements in BMI, fasting glucose, and lipid profiles (All *p* < 0.05). Moreover, delayed recall and immediate recall scores demonstrated a significant increase (All *p* < 0.05). VBM identified gray matter volume increases in left hippocampus, left thalamus, left red nucleus, and left substantia nigra, with left thalamic gray matter volume changes significantly negative correlation with changes of immediate recall scores (*r* = −0.512, *p* = 0.01).

**Conclusion:**

The 1-month eTRE intervention improved metabolic health, reduced brain aging, and enhanced memory in males with MetS. These benefits were associated with structural changes in the brain, indicating that even a short-term eTRE intervention serve as an effective strategy to promote brain health in individuals with MetS.

## 1 Introduction

MetS is a risk factor for various diseases, including cardiovascular disease, cerebrovascular disease, type 2 diabetes, chronic kidney disease, and neurodegenerative disorders such as Alzheimer’s disease, Parkinson’s disease, vascular dementia, and mild cognitive impairment, all of which are closely associated with accelerated brain aging and cognitive decline (Haas et al., 2025). Epidemiological evidence shows that men with MetS have a higher risk of cognitive decline and dementia than age-matched women (Ng et al., 2016).

Brain aging refers to a series of progressive changes associated with cognitive dysfunction and structural degeneration, it serves as an important indicator for evaluating overall brain health, and the decline in memory ability is the most obvious sign of brain aging (Kolenic et al., 2018). Importantly, recent studies have demonstrated that MetS leads to increased brain age and cognitive decline, particularly impairing memory function (Haas et al., 2025; Qureshi et al., 2024). The potential mechanisms include central insulin resistance induced by MetS, which disrupts hippocampal synaptic plasticity and impairs memory consolidation (Malin et al., 2022). Meanwhile, impaired glucose metabolism, elevated oxidative stress, and persistent neuroinflammation further damage neuronal function (Yan et al., 2020; Qureshi, Collister, et al., 2024). Chronic systemic and central inflammation caused by MetS can activate microglia and astrocytes, promoting neuronal injury and memory deterioration (Huang et al., 2024). At the cellular level, MetS-induced metabolic stress impairs mitochondrial function, increases the production of reactive oxygen species (ROS), and disrupts energy metabolism, leading to neuronal damage, synaptic instability, and decreased memory capacity (Rhea et al., 2023). In addition, MetS can cause cerebrovascular dysfunction and microvascular injury, reducing cerebral blood flow and compromising the integrity of the blood–brain barrier, thereby accelerating brain aging (Qureshi et al., 2024). Finaly structural MRI studies have also shown that patients with MetS exhibit reduced volumes in several subcortical regions, indicating structural brain impairment associated with memory integrity (Cakir et al., 2024).

MRI-based brain age estimation predicts an individual’s brain age from gray matter, white matter, and cerebrospinal fluid volumes, with gray matter volume (GMV) being the strongest contributor to prediction accuracy (Teruya et al., 2021). A higher Brain age gap (BAG) was significantly associated with reduced episodic memory and working memory scores in a community adult sample (Wrigglesworth et al., 2022). Additionally, a study about older adults shows that, greater BAG was linked not only to lower fluid intelligence and working memory but also to higher blood glucose levels, hypertension and unfavorable lifestyle factors (Jawinski et al., 2022). These findings underscore the utility of MRI-based brain age estimation as a sensitive marker of brain health and cognitive aging.

Time-Restricted Eating (TRE) is a lifestyle intervention that can effectively improve MetS, it only requires eating within a specified time window, without the need for caloric restriction (Ezpeleta et al., 2024). Studies have shown that TRE can enhance brain-derived neurotrophic factor (BDNF) signaling, which is crucial for cognitive function, neuroplasticity, and antidepressant effects (Currenti et al., 2021). In addition, TRE promotes autophagy in neural cells by regulating metabolic pathways, helping to remove damaged cellular components and maintain neural health (Helbling et al., 2024). Early time-restricted eating (eTRE) is a dietary regimen that confines daily calorie intake to an early time window, typically from morning to mid-afternoon, aligning food consumption with circadian rhythms to improve metabolic health (Bohlman et al., 2024; Elortegui Pascual et al., 2023). eTRE may also improve sleep quality, particularly in individuals with circadian rhythm disturbances (Bohlman et al., 2024). However, it remains uncertain whether eTRE can mitigate brain aging through structural modifications in the brain and, in turn, exert beneficial effects on memory function.

Based on previous research and observations, we conducted a 1-month eTRE intervention in males with MetS, we hypothesized that after the intervention, participants would show improvements in metabolic profiles and memory performance, a reduction in MRI-based brain age, and identify specific brain regions showing alterations associated with memory function.

## 2 Materials and methods

### 2.1 Dataset and participants

23 Maless were recruited from the Endocrinology Outpatient Department of Xi’an Daxing Hospital, hospital announcements, and online advertisements. All the participants signed the informed consent form. Psychiatric status was screened at baseline using clinical history and standardized questionnaires to rule out severe mental illnesses, including the Hamilton Anxiety Scale, Beck Depression Inventory, Patient Health Questionnaire-9, and the 12-Item Short Form Health Survey, which assesses overall mental and physical health status.

1. Inclusion Criteria:

1. Age: from 25 to 55 years.
2. No eating disorder.
3. No severe mental illnesses.
4. Voluntarily sign the informed consent form.
5. Maintaining stable weight (weight change <10%) in the 3 months prior to the study.
6. If participants are taking hypoglycemic, antihypertensive, lipid-lowering, and cardiovascular medications, they are not allowed to adjust the dose during the intervention period.
7. Clinically diagnosed with MetS, defined as the presence of at least three of the following five abnormalities, according to the National Cholesterol Education Program Adult Treatment Panel III (NCEP ATP III) criteria (Alberti et al., 2009).
  - Waist circumference for men ≥90 cm, for women ≥85 cm;
  - Fasting triglycerides ≥1.7 mmol/L;
  - Fasting high-density lipoprotein cholesterol <1.04 mmol/L;
  - Systolic/diastolic blood pressure ≥130/85 mmHg, and (or) those who have been diagnosed with hypertension and are being treated;
  - Fasting blood glucose ≥6.1 mmol/L and (or) 2-h postprandial blood glucose ≥7.8 mmol/L, and (or) those who have been diagnosed with diabetes and are being treated.

2. Exclusion Criteria:

1. Night shift workers.
2. History of weight loss surgery.
3. MRI examination contraindications.
4. Presence of neurological disorders or brain dysfunction.
5. History of head injury or brain lesions.
6. Addiction to tobacco or alcohol.
7. Exclusion related to safety concerns.
8. Inability to adhere strictly to the study protocol.
9. History of endocrine disorders or obesity resulting from single-gene mutations, including but not limited to hypothalamic obesity, pituitary obesity, hypothyroid obesity, Cushing’s syndrome, insulinoma, acromegaly and hypogonadism.
10. Experienced two or more hypoglycemic events in the 6 months before screening, defined as blood glucose <2.8 mmol/L, or blood glucose not reaching <2.8 mmol/L but with evident hypoglycemic symptoms.
11. History of significant illness or related diseases, such as inflammatory diseases, rheumatic autoimmune diseases, adrenal diseases, malignant tumours, type 1 diabetes, liver cirrhosis, chronic kidney disease, acquired immunodeficiency syndrome, eating disorders, mental disorders, and adverse cardiovascular events.
12. Severe gastrointestinal diseases or gastrointestinal surgeries within the past 12 months, actively participating in weight loss programs, using medications that affect weight or energy balance, currently participating in other weight management programs, or receiving prescribed diets due to specific diseases or taking any medications that affect appetite.

### 2.2 MRI data acquisition

All subjects will undergo MRI scans using 3T MRI (Prisma, SIEMENS MAGNETOM, Germany) with 32 head chanle, which include T1-Magnetization Prepared Rapid Gradient Echo (T1-MPRAGE) and Axial T2WI(performed to exclude intracranial lesions). The specific sequence order and parameters are as follows:

T1-MPRAGE: scan time = 3 min 9 s, number of slices = 176, voxel size = 1.0 mm × 1.0 mm × 1.0 mm, repetition time (TR) = 1,540 ms, echo time (TE) = 2.99 ms, flip angle (FA) = 9°, field of view (FOV) = 224 mm × 224 mm, and image resolution = 224 × 224.

T2WI: scan time = 1 min 2 s, slicens = 22, voxel size = 0.75 mm × 0.6 mm × 5.5 mm, repetition time (TR) = 4530 ms, echo time (TE) = 109 ms, flip angle (FA) = 150°, field of view (FOV) = 194 mm × 230 mm, and image resolution = 259 × 224.

### 2.3 Brain age prediction

All 3D T1-weighted images were preprocessed using Statistical Parametric Mapping (SPM12, http://www.fil.ion.ucl.ac.uk/spm/software/spm12) implemented on MATLAB R2023a. The raw DICOM images were converted into NIFTI-1 further processing. Preprocessing steps included:

The NIFTI images were first denoised and bias-field corrected to improve the signal-to-noise ratio and to correct intensity inhomogeneities in uniform regions. Subsequently, skull stripping was performed to remove non-brain tissues, after which the images were segmented into gray matter, white matter, and cerebrospinal fluid. Voxel values were then modulated using the Jacobian determinants to preserve local tissue volumes, and finally resampled to an isotropic voxel size of 1.5 mm^3^ (Ashburner, 2007).

For brain age prediction, we employed the publicly available brainageR software package (https://github.com/james-cole/brainageR) (Clausen et al., 2022). This tool uses a machine learning approach trained on 3,477 healthy individuals across the adult lifespan (Cole and Franke, 2017). This package further processed the gray matter GM images. Specifically, the preprocessed GM maps were smoothed with a 4-mm full-width at half-maximum (FWHM) Gaussian kernel to enhance the signal-to-noise ratio and reduce inter-individual anatomical variability. The smoothed voxel-wise GM volumes were then used as input features for a trained regression model. Finally, the algorithm generated a predicted brain age for each participant.

The Brain Age Gap (BAG) was computed for each individual as:

$${GAP}_{i}={Brain\;age}_{i}-{Chronological\;age}_{i}$$

${Brain\;age}_{i}$ where represents the predicted brain age and ${Chronological\;age}_{i}$ is the actual age of participants. A positive BAG indicates accelerated brain aging, whereas a negative BAG reflects delayed brain aging (Cole et al., 2018).

### 2.4 VBM analysis

NIFTI images were preprocessed using the Computational Anatomy Toolbox (CAT12, https://neuro-jena.github.io/cat/). Images were segmented into gray matter, white matter, and cerebrospinal fluid, followed by modulation of voxel values using the Jacobian determinants derived from spatial normalization to preserve local tissue volumes. The normalized gray matter maps were resampled to an isotropic voxel size of 1.5 mm^3^. For voxel-based morphometry analyses, the modulated gray matter maps were smoothed with an 8-mm full-width-at-half-maximum Gaussian kernel prior to statistical analysis. To control for inter-individual differences in head size, total intracranial volume was included as a covariate in the statistical model (Gaser et al., 2024).

### 2.5 Study design and eTRE procedures

Before the intervention began, all participants were asked to maintain their regular diet and exercise habits for 1-month to stabilize their weight. During the 1-month intervention period, all participants were instructed to consume their calories between 8 a.m. and 4 p.m. each day, fasting from 4 p.m. until 8 a.m. During the eating window, participants could eat freely without any restrictions on the quantities or types of food, while maintaining their usual dietary patterns. In 16 h fasting window, participants were recommended to drink plenty of water, and unlike other similar TRE studies, we prohibited participants from consuming any beverage during the fasting period, including both sugar-free and fat-free beverages and tea. We provide detailed guidance on time-restricted eating to participants, including specific timing of the eating window (Currenti et al., 2021).

### 2.6 Quality control

To monitor all participants during the fasting window, participants used a Continuous Glucose Monitor (CGM, from WeiTai Health Medical Technology Co., LTD China Shanghai), which was connected to their mobile phones and the endocrinologist phones by an APP for real-time monitoring. If any participant ate outside the fasting window, the endocrinologist would receive alerts indicating elevated blood glucose levels, and immediately Contact the participants keep to follow the dietary rules.

A separate social media group was established for each participant, consisting of the participants, one endocrinologist, one nutrition nurse, and three radiologists. Physicians provided daily supervision and encouragement within the group, while participants were required to upload photos of their meals each day. All physicians in the group responded to participants’ questions in real time, offering continuous support and motivation to enhance adherence and confidence in the intervention.

Comprehensive data quality control and adherence verification procedures were implemented throughout the study. Detailed descriptions of the CGM configuration, spike detection algorithm, calibration procedures, and adherence feedback mechanisms are provided in the Supplementary Methods.

### 2.7 Clinical data

Baseline assessments included demographic and clinical variables such as sex, age, height, weight, educational level, and history of smoking and alcohol consumption. Anthropometric measurements were obtained using a body composition analyzer (InBody 770, InBody, Seoul, Korea), which provides a precision of ±0.1 kg for body weight and ±0.5 cm for height via its integrated stadiometer. Measurements were performed with participants barefoot and wearing light clothing. Body mass index (BMI) was calculated as weight in kilograms divided by height in meters squared (kg/m^2^) and evaluated at both baseline and after the 1-month intervention.

Venous blood samples were collected at the Clinical Laboratory of Xi’an Daxing Hospital in the morning (07:40–09:00 h) following an overnight fast beginning at 20:00 h, at baseline and again after 1 month. Biochemical analyses included triglycerides, total cholesterol, high-density lipoprotein cholesterol, low-density lipoprotein cholesterol, fasting plasma glucose, and fasting insulin. Indices of insulin resistance and sensitivity were derived, including the homeostatic model assessment of insulin resistance (HOMA-IR) and the quantitative insulin sensitivity check index (QUICKI), calculated from fasting plasma glucose and FINS values. Additionally, the following indices were calculated:

$$\text{HOMA}-\text{IR}=\frac{FPG\left(mmol/L\right)\times FINS\left(\mu U/mL\right)}{22.5}$$

$$\text{QUICKI}=\frac{1}{logFPG\left(mmol/L\right)+logFINS\left(\mu U/mL\right)}$$

### 2.8 Psychological scales

Memory function was assessed using the Rey Auditory Verbal Learning Test (RAVLT), originally developed by Rey (Loring et al., 2023). In this study, we applied the validated Chinese version of RAVLT (Dong et al., 2023). The test evaluates verbal learning and memory across two main domains: immediate recall and delayed recall.

Immediate recall was measured by the sum of the first five learning trials and the additional immediate recall trials, while delayed memory was assessed via the scores of the delayed recall trials. Recognition memory was also recorded. Higher scores indicate better memory performance (Jannati et al., 2024).

### 2.9 Statistical analysis

Statistical analyses were conducted using SPSS Statistics software (IBM Corp., Armonk, NY, United States, version 29.0). Continuous variables were expressed as mean ± standard deviation (SD).

We used paired *t*-tests for continuous variables and Pearson’s χ^2^ tests for categorical variables to compare differences between baseline and 1-month eTRE, measurements, including demographic characteristics, BAG, and clinical data such as body weight, BMI, fasting glucose, and blood lipid parameters. Pearson correlation analysis was performed to evaluate associations between BAG and RAVLT scores.

We performed voxel-wise paired t-tests to compare GMV between baseline and 1-month after eTRE. Brain regions showing significant GMV changes were defined as regions of interest (ROIs). The mean GMV of ROIs was extracted for each participant, and Pearson correlation analyses were subsequently conducted to evaluate the relationships between GMV and the scores of different dimensions of the RAVLT. If multiple brain regions showed significant changes, the correlation results were corrected for multiple comparisons using the Bonferroni method.

### 2.10 Ethics statement

The study was performed in accordance with the ethical standards of the Declaration of Helsinki World Medical Association (1964) and its subsequent amendments. All procedures involving human participants were reviewed and approved by the Medical Ethics Committee of Xi’an Daxing Hospital (Approval Number: KY 2024-025). Written informed consent was obtained from all participants.

## 3 Result

### 3.1 Demographics and clinical information

**FIGURE 1:**
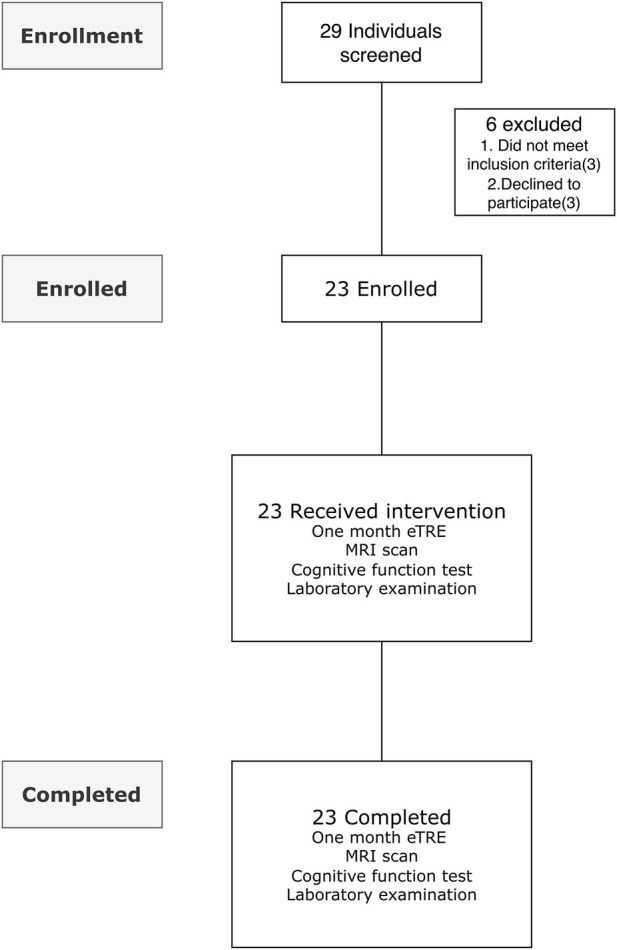
Experimental flowchart.

A total of 23 participants were enrolled and successfully completed the study protocol (Figure 1). Following the 1-month eTRE intervention, participants RAVLT showing a significant increase (*p* < 0.01). Significant reductions were observed in body weight, BMI, TD, FPG, FINS, HOMA-IR, while insulin sensitivity, as indicated by QUICKI, significantly improved (all *p* < 0.05). Only one participant reported regular alcohol consumption, with an average intake of 200 mL per day, show in Table 1.

**TABLE 1:** Baseline characteristics, and changes in metabolic risk factors and memory performance after a 1-month eTRE intervention in men with MetS (n = 23).

​	Baseline	1-month eTRE	*t*	*P* value
Age (years)	35.42 ± 6.32	NA	NA	NA
Education (years)	15.51 ± 0.70	NA	NA	NA
Smoking, n (%)	7 (30.43)	NA	NA	NA
Regular alcohol, n (%)	1 (0.04)	NA	NA	NA
Weight (kg)	95.57 ± 12.95	91.20 ± 11.96	−9.03	<0.001*
BMI (kg/m^2^)	31.42 ± 4.19	29.98 ± 3.92	−9.15	<0.001*
TD (mmol/L)	2.96 ± 1.96	2.09 ± 1.40	−5.19	<0.001*
TC (mmol/L)	4.72 ± 1.06	4.55 ± 0.87	−1.83	0.08
HDL-C (mmol/L)	0.94 ± 0.17	0.95 ± 0.19	0.15	0.88
LDL-C (mmol/L)	2.70 ± 0.78	2.79 ± 0.72	0.74	0.47
FPG (mmol/L)	6.54 ± 1.98	5.88 ± 1.05	−2.25	0.011*
FINS(mmol/L)	6.52 ± 4.61	3.95 ± 2.38	−3.00	<0.001*
HOMA-IR	6.52 ± 4.61	3.95 ± 2.38	−3.00	0.007*
QUICKI	0.30 ± 0.02	0.32 ± 0.03	3.34	0.003*
RAVLT test				
Delayed recall (points)	9.43 ± 2.79	11.30 ± 2.69	3.71	0.001*
mmediate recall (points)	45.35 ± 7.54	54.09 ± 9.35	5.14	<0.001*
Interference recall (points)	5.74 ± 1.71	5.56 ± 2.01	−0.33	0.74

BMI, body mass index; TD, triglycerides; TC, total cholesterol; HDL-C, high density lipoprotein cholesterol; LDL-C, Low-Density Lipoprotein Cholesterol; FPG, fasting plasma glucose; FINS, fasting insulin; HOMA-IR, homeostasis model assessment of insulin resistance; QUICKI, quantitative insulin sensitivity check index; RAVLT, Rey Auditory Verbal Learning Test. All t-values were derived from two-tailed tests, with statistical significance set at *p* < 0.05 (*: *P* < 0.05).

### 3.2 BAG analyse

**FIGURE 2:**
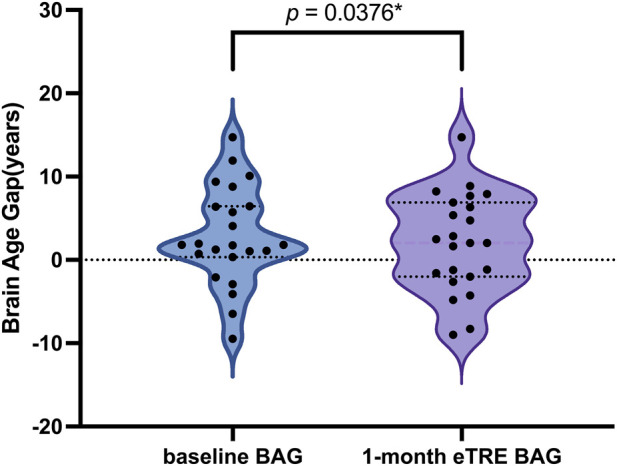
Effect of 1-month eTRE on BAG in males with MetS. After the intervention, BAG was significantly reduced (t = −0.760, *P* = 0.0376).

**FIGURE 3:**
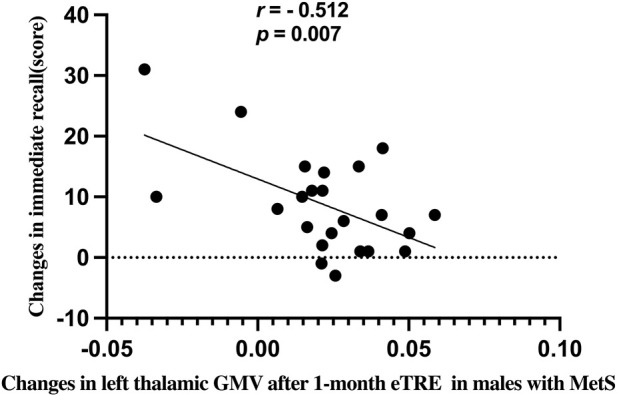
After the 1-month eTRE intervention, a negative correlation was observed between left thalamic GMV changes and immediate recall score changes in males with MetS (*r* = −0.512, *p* = 0.007, *Bonferroni* corrected).

After a 1-month eTRE intervention in male patients with MetS, the BAG showed a statistically significant reduction (t = −0.760, p < 0.05). The mean BAG decreased from 2.794 ± 5.867 at baseline to 2.034 ± 5.884 after the intervention, corresponding to an average reduction of 0.760 years (Figures 2, 3).

### 3.3 VBM analyse

After 1-month eTRE intervention in males with MetS, GMV increased in the left hippocampus, the left red nucleus, the left substantia nigra and the left thalamus (Table 2).

**TABLE 2:** Changes in GMV after 1-month eTRE intervention in males with MetS (n = 23).

Brain region	BA	MNI Coordinates			Peak intensity	Voxels
		X	Y	Z		
Left Hippocampus	BA30	−17	−26	−11	4.64	19
Left Thalamus	-	−12	−30	−3	4.53	165
Left Red nucleus	-	−6	−23	−9	4.95	8
Left Substantia nigra	-	−15	−23	−9	4.09	14

### 3.4 Correlation between changes in GMV and RAVLT score in males with MetS after 1-month of eTRE

Among the four brain regions showing changes in GMV, only the left thalamus exhibited a significant correlation with immediate recall score.

## 4 Discussion

After a 1-month eTRE intervention, males with MetS showed significant reductions in body weight, BMI, TG, FPG, FINS, and HOMA-IR, accompanied by increases in QUICKI and RAVLT scores. Furthermore, the thalamus–hippocampal memory circuit and several reward-related regions were identified as the brain areas contributing most to the improvement in brain age. Moreover, we found that changes in the GMV of the left thalamus were significantly correlated with changes in immediate recall scores. These findings indicate that even a 1-month eTRE intervention can significantly improve metabolic function, enhance memory performance, and slow brain aging, with the thalamus potentially serving as a key region underlying memory improvement.

In our study, the 1-month eTRE intervention was found to slow brain aging in males with MetS, and several potential mechanisms may underlie this effect. First, TRE can improve cerebrovascular and metabolic regulation by enhancing endothelial function and neurovascular coupling, thereby increasing cerebral perfusion and reducing vascular stress and inflammation (Da C Pinaffi-Langley et al., 2024). Second, TRE may enhance neurotrophic support by increasing the expression of brain-derived neurotrophic factor, which promotes neurogenesis and synaptic plasticity, thereby strengthening brain resilience (Ezpeleta et al., 2024). Third, TRE facilitates the clearance of damaged cellular components through autophagy and optimizes mitochondrial function, thereby protecting the brain from oxidative stress and inflammation (Lettieri-Barbato et al., 2018; Currenti et al., 2021; Helbling et al., 2024). These processes collectively preserve neural integrity and slow the trajectory of brain aging. However, the improvements observed in our study may not be attributable solely to eTRE *per se*. Shortening the eating window may also lead to a spontaneous reduction in caloric intake, which has been documented in other TRE studies and shown to contribute to weight loss and metabolic improvement (Jamshed et al., 2022). Because daily caloric intake was not quantitatively assessed in the present study, we were unable to disentangle the independent effects of eating-window timing from those of caloric reduction. It is therefore more likely that the observed metabolic and brain structural changes reflect the combined influence of circadian alignment induced by eTRE and concurrent reductions in energy intake.

We conducted only a 1-month intervention in males with MetS, yet participants already showed improvements in memory performance, including both immediate recall and delayed recall. A recent animal study has shown that even just 4 days of a high-fat diet can impair memory function, the mechanism being the induction of hyperactivity in glucose-inhibited cholecystokinin-expressing interneurons (CCK-INs) within the dentate gyrus (DG), accompanied by elevated phosphorylation of pyruvate kinase M2 (PKM2) and reduced hippocampal glucose availability (Landry et al., 2025). This finding suggests that metabolic stress directly disrupts hippocampal neural circuits essential for memory encoding. So it is plausible to suppose that a short-term intervention may counteract these effects by restoring glucose homeostasis, reducing PKM2 activity, and normalizing neuronal excitability in hippocampal memory networks. Thus, even a 1-month eTRE intervention may be sufficient to reverse early metabolic dysregulation, thereby improving memory performance and preventing cognitive decline in males with MetS.

Notably, alterations in left thalamic GMV showed a negative correlation with changes in immediate recall performance. This relationship suggests that thalamic structural remodeling may not linearly correspond to functional recovery, possibly due to the thalamus’s complex structure and diverse nuclei, each contributing differently to memory processing (Aggleton et al., 2010). Similar paradoxical patterns have been observed in other brain regions, such as the hippocampus and prefrontal cortex, where early volumetric increases precede or even inversely relate to behavioral gains (Zatorre et al., 2012; Tymofiyeva and Gaschler, 2021; Wenger et al., 2017). This pattern suggests that structural remodeling may initially represent compensatory or adaptive reorganization before functional optimization occurs over time. Overall, these findings indicate that structural and functional adaptations following eTRE likely occur asynchronously.

In our study, we observed that structural changes in the thalamus were significantly associated with improvements in immediate recall. It is likely attributable to the functional specialization of different thalamic nuclei in memory processing. Immediate recall and working memory are predominantly supported by the reciprocal circuitry between the dorsolateral prefrontal cortex and the mediodorsal thalamic nucleus, which is known to sustain active maintenance of information through persistent delay-period activity and prefrontal–thalamic synchrony (Watanabe and Funahashi, 2012). In contrast, delayed recall consolidation and delayed recall rely more heavily on the anterior thalamic nuclei (ATN) and their interactions with the hippocampal formation. The ATN–hippocampal network contributes to memory encoding and retrieval through theta–gamma coupling and large-scale circuit integration, processes that are well documented in recent human and animal studies (Aggleton et al., 2010; Hwang et al., 2022; Sweeney-Reed et al., 2021). Although immediate recall and delayed recall both performance improved following the eTRE intervention, only immediate recall was associated with measurable structural changes in the thalamus. Several factors may account for this pattern. First, the sample size in the present study may not have been sufficient to detect subtle structural changes within the ATN–hippocampal system. Second, given the structural and functional heterogeneity of the thalamus, different nuclei may exhibit distinct recovery trajectories under metabolic or lifestyle interventions such as eTRE. It is therefore plausible that nuclei supporting short-term memory undergo more rapid or more detectable structural adaptation, whereas nuclei involved in long-term memory processes may require larger samples or longer intervention durations to reveal similar structural–behavioral coupling. Taken together, our finding underscores the nucleus-specific role of the thalamus in memory and suggests that early structural plasticity within the memory-related circuitry may emerge first during immediate recall improvement, whereas slower changes in nuclei supporting delayed recall cannot be ruled out.

## 5 Limitations

Our study has several limitations. First, the small sample size calls for a larger cohort in future research to enhance the reliability and robustness of our findings. Second, the study included only males with MetS; future studies should include femaless to improve the generalizability of the results. Third, although we observed improvements in brain structure and metabolic outcomes after 1 month of eTRE, these effects likely reflect a combination of mechanisms, including improved metabolic rhythm alignment due to time-restricted eating and a concomitant reduction in caloric intake resulting from a shortened eating window. The present study was not designed to disentangle the relative contributions of these factors. Accordingly, future work will incorporate precise and systematic assessments of daily caloric intake to distinguish timing-dependent effects from those driven by caloric restriction. Finally, the intervention and follow-up period were relatively short; ongoing longitudinal follow-up will allow us to evaluate the persistence and long-term effects of eTRE on brain structure and cognitive function.

## 6 Conclusion

One-month eTRE may serve as an effective intervention to protect brain health in individuals with MetS. We found that eTRE Mitigated brain aging, improved memory performance. Highlighting a potential neural mechanism underlying the cognitive benefits of eTRE.

## Data Availability

The raw data supporting the conclusions of this article will be made available by the authors, without undue reservation.
