# Supplementary material for: One-month early time-restricted eating mitigates brain aging and enhances memory in males with metabolic syndrome: an MRI structural study
Source: Front Aging. 2026 Feb 18;7:1752738. doi: 10.3389/fragi.2026.1752738 (PMC12957201; doi:10.3389/fragi.2026.1752738)
Supplement: Supplementary file 1 [file Supplementaryfile1.docx]

# ****Supplementary Methods****

## CGM-Based Detection of Out-of-Window Ingestion Events in Time-Restricted Eating (TRE)

1. Continuous Glucose and Behavioral Monitoring

All participants were equipped with a continuous glucose monitoring system (CGM; WeiTai Health Medical Technology Co., Ltd., Shanghai, China) for real-time glucose tracking, and a Huawei Band 8 smart band to monitor sleep and physical activity. Participants were instructed to wear both devices continuously (24 h/day) throughout the 4-week intervention to ensure data completeness and accuracy.

The CGM system was configured to trigger hypoglycemia alerts when interstitial glucose levels fell below 3.9 mmol/L. Across the cohort, hypoglycemia alerts accounted for less than 1% of total recordings, with approximately half of participants exhibiting fewer than 0.1% alerts.

Sleep duration and physical activity remained stable across participants, except for one individual who demonstrated greater intra-individual variability without compromising overall data integrity.

2. Detection of Out-of-Window Ingestion Events

2.1 Eating Window and Deviation Criteria

Participants followed a fixed early time-restricted eating (eTRE) schedule from 08:00 to 16:00 daily. Any caloric intake outside this window was considered a protocol deviation.Before the intervention, each participant underwent a two-day baseline CGM recording under habitual eating conditions to characterize individual glucose dynamics.

2.2 Automated Spike Detection Algorithm

An “out-of-window ingestion” event was defined as a glucose excursion meeting all of the following conditions:Amplitude Criterion: a rise of ≥0.8 mmol/L above the preceding baseline;Rate-of-Change Criterion: a rate of increase >0.02 mmol/L/min (≈0.3 mmol/L within 15 minutes).These thresholds were determined from prior literature showing that healthy individuals typically exhibit postprandial excursions of 2.5–3.0 mmol/L (Freckmann et al., 2024) and supported by CGM-based meal detection studies(He et al., 2022), which reported optimal sensitivity for amplitude thresholds between 0.5–1.0 mmol/L and slopes of 0.015–0.03 mmol/L/min.

2.3 Duration and Boundary Tolerance

To reduce false-positive detection due to transient fluctuations, a glucose elevation was considered a valid out-of-window ingestion event only if:
(i) the elevation lasted ≥30 minutes, and
(ii) occurred >30 minutes away from either boundary of the eating window (08:00 or 16:00).

2.4 Day Classification and Adherence Threshold

Any spike fulfilling these criteria and not associated with a self-reported meal or physical activity episode was labeled as an out-of-window ingestion event.A non-adherent day was defined as any day containing ≥1 such event. Participants with ≥80% of days free from out-of-window ingestion were categorized as adherent(Pan et al., 2024); others were categorized as non-adherent. This operational threshold aligns with adherence definitions used in recent TRE–CGM feasibility studies.

2.5 Calibration and Sensitivity Validation

A 7–14-day pilot calibration phase was conducted before the main intervention to optimize event detection parameters for the specific CGM system. During calibration, CGM-derived glucose traces were matched with participants’ self-reported meal logs to evaluate combinations of amplitude thresholds (0.6, 0.8, 1.0 mmol/L) and rate thresholds (0.015–0.03 mmol/L/min). Receiver operating characteristic (ROC) analysis was used to identify the parameter combination that maximized both sensitivity and specificity. The optimal set was adopted for all subsequent analyses (see Supplementary Tables).This calibration and machine learning–assisted detection approach followed established CGM analytical frameworks(Wang et al., 2021).

2.6 Participant Feedback and Adherence Support

According to the above criteria, all participants achieved ≥80% adherence. A total of 38 out-of-window episodes were detected: 20 associated with physical activity and 18 due to dietary non-adherence.

Each participant was provided with an individualized online platform for continuous data upload, communication, and personalized feedback. The multidisciplinary team (MDT) reviewed daily logs of diet, sleep, and physical activity, offering real-time behavioral and dietary counseling. In addition to adherence support, the research team also responded to hypoglycemia alerts in real time, contacting participants to confirm symptoms and provide individualized dietary guidance when necessary.

Participants completed short daily questionnaires on hunger and emotional state, which facilitated timely psychological and behavioral adjustments. This integrated feedback system helped maintain high adherence and ensured participant privacy throughout the intervention.

Manoogian, E.N.C., Chow, L.S., Taub, P.R., Laferrère, B., Panda, S., 2022. Time-restricted eating for the prevention and management of metabolic diseases. Endocrine Reviews 43, 405–436. https://doi.org/10.1210/endrev/bnab027

Pan, G., Zhang, J., Sun, Y., Shi, Y., Zhang, F., 2024. Saturation association between serum 25-hydroxyvitamin D levels and mortality in elderly people with hyperlipidemia: A population-based study from the NHANES (2001-2016). Front. Endocrinol. 15, 1382419. https://doi.org/10.3389/fendo.2024.1382419

Wang, Y., Yin, X., Zhang, Z., Li, J., Zhao, W., Guo, Z.V., 2021. A cortico-basal ganglia-thalamo-cortical channel underlying short-term memory. Neuron 109, 3486-3499.e7. https://doi.org/10.1016/j.neuron.2021.08.002
